# Liquid biopsy: blood‐based analyses of circulating cell‐free DNA in xenografts

**DOI:** 10.15252/emmm.202216326

**Published:** 2022-07-29

**Authors:** Isabel Heidrich, Klaus Pantel

**Affiliations:** ^1^ Department of Tumor Biology University Medical, Center Hamburg‐Eppendorf Hamburg Germany; ^2^ Department of Dermatology and Venereology, Skin Cancer Center University Hospital Hamburg‐Eppendorf (UKE) Hamburg Germany

**Keywords:** Cancer

## Abstract

The liquid biopsy concept has been introduced for circulating tumor cells more than 10 years ago (Pantel & Alix‐Panabieres, 2010) and rapidly extended to cell‐free DNA released from tumor cells (ctDNA; Lo *et al*, 2021) and other tumor‐derived products such as circulating cell‐free RNA (noncoding and messenger RNA), extracellular vesicles, or tumor‐educated platelets (Alix‐Panabières & Pantel, 2021). In this issue of *EMBO Molecular Medicine*, the report of Sauer *et al* (2022) demonstrates the feasibility of longitudinal monitoring of disease burden and response using ctDNA from dried blood spots in xenograft models.

## Graphical abstract

I. Heidrich and K. Pantel discuss the study by Sauer *et al* (in this issue of *EMBO Mol Med*) that demonstrates the feasibility of longitudinal monitoring of disease burden and response using ctDNA from dried blood spots in xenograft models.

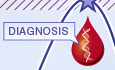

Patient‐derived xenograft (PDX) mice are frequently used as models to study new treatment approaches for human cancers. Sauer *et al* (2022) developed shallow WGS (sWGS) of ctDNA from serial dried blood spot (DBS) samples and showed that copy number changes are detected over multiple time points and DBS ctDNA recapitulates the biological features of ctDNA in patients. Sequential DBS ctDNA accurately predicted treatment response and disease outcome in PDX mouse models. Their approach enabled sequential blood sampling and sWGS‐based detection of ctDNA over time from minute volumes of whole blood (~50 μl) in clinically relevant animal models.

Besides its use in xenograft mice models, this new approach has important implications for liquid biopsy analyses in humans. Dried blood spot samples can be taken easily by the cancer patient or individual at risk of developing cancer at home and sent to a central laboratory for ctDNA‐based analysis. Self‐sampling of blood opens therefore a new avenue for liquid biopsy diagnostics. In addition, the DBS method can now also be applied to understand the biology behind ctDNA as a biomarker. To date, we still do not fully understand why even some metastatic tumors are poor shedders, which limits the use of ctDNA. This heterogeneity occurs between and within different tumor entities and cannot be easily predicted. The tumor microenvironment might play a role; primary or metastatic lesions in the brain usually show lower ctDNA concentrations than tumors in other organs. Moreover, the biological role of ctDNA encapsulated in extracellular vesicles is still debated and the hypothesis that ctDNA (or cell‐free DNA in general) might play an important functional role in disease evolution. Finally, the concentration of ctDNA depends on the background of cell‐free non‐cancer DNA, which can also change over time due to disease conditions (e.g., renal failure) or therapies inducing massive apoptosis of normal tissue (e.g., chemotherapy).

The main source of ctDNA is apoptotic tumor cells, but active secretion through encapsulation in extracellular vesicles has been also demonstrated (Lo *et al*, 2021). In particular, high‐resolution, scalable blood‐based detection methods of ctDNA have been developed over the past decade, and peripheral blood has become the key fluid for LB analysis (Alix‐Panabières & Pantel, 2021; Fig 1). Recently, tracking tumors by liquid biopsy analyses has been highlighted as a milestone discovery of the past 20 years (Romero, 2020). The benefit for cancer patients consists in the fact that ctDNA blood analyses—as opposed to repetitive tissue biopsies—are minimal or noninvasive and more sensitive than conventional imaging. ctDNA has already been used in numerous clinical trials, and its clinical utility is currently under investigation, with promising results in clinical studies on cancer patients (Tie *et al*, 2022). Clinical applications include early cancer detection, improved cancer staging, early detection of minimal residual disease and relapse, real‐time monitoring of therapeutic efficacy and detection of therapeutic targets and resistance mechanisms (Alix‐Panabières & Pantel, 2021; Fig 1).

**Figure 1:**
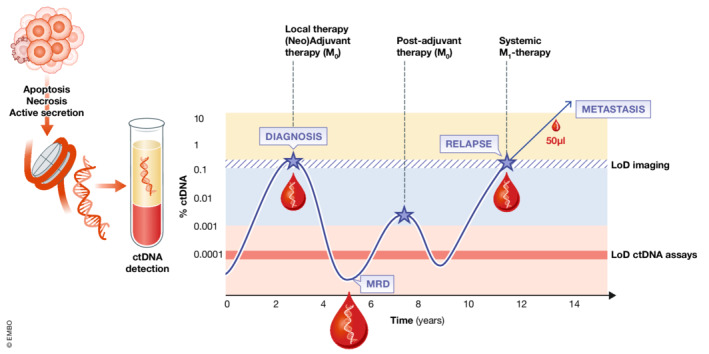
Monitoring of ctDNA in cancer patients during tumor evolution ctDNA analysis is based on the identification of tumor‐specific aberrations or epigenetic marks on circulating cell‐free DNA in blood plasma samples. It enables the development of new methods for early detection of primary cancer or disease relapse, monitoring the efficacy of cancer therapies, and determining therapeutic targets and resistance mechanisms to adapt therapy to the specific needs of an individual patient. The advantage is the noninvasive collection of tumor material and sequential monitoring of ctDNA in blood samples over time. However, blood volume is a critical factor in liquid biopsy analyses determining assay sensitivity. The size of the blood drops indicates the blood volumes required at different disease stages depending on the respective tumor burden in the cancer patient and the amount of ctDNA available in the blood sample.

Personalized diagnostic and treatment strategies in cancer are highly based on the individual characteristics associated with malignant transformation and progression (Hahn *et al*, 2021). To date, most patients are categorized at initial diagnosis, and treatment remains based on this initial classification while the tumor might undergo fundamental genotypic and functional changes. Recent research has confirmed that natural and therapy‐induced selective pressure leads to clonal dynamics in cancers, leading to considerable heterogeneity and treatment resistance (Keller & Pantel, 2019; Marine *et al*, 2020). To monitor these complex dynamic processes in cancer patients, sequential monitoring of tumor composition in individual cancer patients by repeated ctDNA‐based blood tests is required to adapt therapies to these changes (Fig 1).

In contrast to the plethora of clinical studies using ctDNA as a biomarker, there is a lack of experimental studies in mice which would greatly enhance our knowledge of the biology of this important biomarker. One reason for this discrepancy is the low concentration of ctDNA and the small volume of blood available for ctDNA‐based analysis, that is, approximately only 1 ml even after heart puncture, which requires sacrificing the animal and precludes sequential monitoring of tumor responses in living mice. In contrast to tumor‐associated RNA species or proteins that are released in high abundancies, cell‐free fragments of tumor‐derived DNA occur at minute amounts in the ocean of cell‐free DNA released from non‐cancer normal cells (e.g., white blood cells). Interestingly, although the tumor specificity of DNA analysis is usually higher than that of RNA or proteins, white blood cells (and probably also other cell types) can harbor tumor‐specific mutations of the KRAS or TP53 genes (and others) in particular in aging individuals (Lo *et al*, 2021).

Before the DBS method can be implemented into clinical practice, future validation studies on larger patient cohorts are required. Moreover, blood volume is a critical factor in liquid biopsy analyses determining assay sensitivity (Fig 1). Most likely, the method will gain clinical relevance in cancer patients with advanced disease (e.g., multiple metastases in distant organs) where the total tumor burden is high and the concentration of ctDNA is far beyond 1% (Lo *et al*, 2021). In contrast, patients with a localized or minimal residual disease after initial therapy usually harbor minute amounts of ctDNA in their blood that are already difficult to detect using regular blood tube volumes of 7–10 ml. The same is true for blood analyses aimed at detecting ctDNA traces for cancer screening of individuals at risk. Nevertheless, the availability of the DBS ctDNA method will allow a denser monitoring of tumor responses to therapy in individual cancer patients with metastatic disease (Fig 1), which is a significant advancement. Especially in patients with advanced metastatic cancer, blood samples are frequently taken to monitor a plethora of important clinical parameters, limiting the amounts available for additional liquid biopsy analyses.

To implement the DBS approach into clinical practice, future interventional clinical studies where clinical decision‐making will be based on the result of the liquid biopsy assay are required. Moreover, assay standardization and quality assurance will be important (Lampignano *et al*, 2020), and international consortia such as the European Liquid Biopsy Society (www.elbs.eu) are now able to fulfill this important task.

## Disclosure and competing interests statement

KP received research support from EU/IMI CANCER‐ID EFPIA and speaker or advisory board honoraria from Agena, Menarini, Novartis, Sanofi, Illumina, Abcam, and Humingbird, and he has the EU patent application Nr. 18705153.7 (PCT/EP2018/054052) pending.
